# Genomic signatures and insights into host niche adaptation of the entomopathogenic fungus *Metarhizium humberi*

**DOI:** 10.1093/g3journal/jkab416

**Published:** 2021-12-04

**Authors:** Natasha Sant′Anna Iwanicki, Ana Beatriz Riguetti Zanardo Botelho, Ingeborg Klingen, Italo Delalibera Júnior, Simeon Rossmann, Erik Lysøe

**Affiliations:** 1 Department of Entomology and Acarology, “Luiz de Queiroz” College of Agriculture (ESALQ/USP), Piracicaba 13418-900, Brazil; 2 Division of Biotechnology and Plant Health, Norwegian Institute of Bioeconomy Research (NIBIO), Ås 1431, Norway

**Keywords:** entomopathogenic fungus, genomic features, specialization, host adaptation

## Abstract

The genus *Metarhizium* is composed of species used in biological control programs of agricultural pests worldwide. This genus includes common fungal pathogen of many insects and mites and endophytes that can increase plant growth. *Metarhizium humberi* was recently described as a new species. This species is highly virulent against some insect pests and promotes growth in sugarcane, strawberry, and soybean crops. In this study, we sequenced the genome of *M. humberi*, isolate ESALQ1638, and performed a functional analysis to determine its genomic signatures and highlight the genes and biological processes associated with its lifestyle. The genome annotation predicted 10633 genes in *M. humberi*, of which 92.0% are assigned putative functions, and ∼17% of the genome was annotated as repetitive sequences. We found that 18.5% of the *M. humberi* genome is similar to experimentally validated proteins associated with pathogen–host interaction. Compared to the genomes of eight *Metarhizium* species, the *M. humberi* ESALQ1638 genome revealed some unique traits that stood out, *e.g.*, more genes functionally annotated as polyketide synthases (PKSs), overrepresended GO-terms associated to transport of ions, organic and amino acid, a higher percentage of repetitive elements, and higher levels of RIP-induced point mutations. The *M. humberi* genome will serve as a resource for promoting studies on genome structure and evolution that can contribute to research on biological control and plant biostimulation. Thus, the genomic data supported the broad host range of this species within the generalist PARB clade and suggested that *M. humberi* ESALQ1638 might be particularly good at producing secondary metabolites and might be more efficient in transporting amino acids and organic compounds.

## Introduction

Historically, the genus *Metarhizium* refers to green-spored asexual insect pathogenic fungi belonging to the Clavicipitaceae family (Kepler *et al.* 2014). It is one of the best characterized and widely studied entomopathogenic fungal genera concerning ecology, evolution, pathogenicity, life history, and genome biology (Lovett and St. Leger 2018). *Metarhizium* species have multifunctional lifestyles, including roles as insect pathogens, plant symbionts, and saprobes (Barelli *et al.* 2016; Vega 2018). Genomic analyses have shown that *Metarhizium* spp. are more closely related to endophytes and plant pathogens than to arthropod pathogens (Gao *et al.* 2011; Hu *et al.* 2014; Stone and Bidochka 2020). The large number of genes for plant degrading enzymes within *Metarhizium* genomes has suggested that they have evolved from fungi that were associated with plants and that the ability to infect and kill insects was a more recently acquired adaptation (Gao *et al.* 2011; Hu *et al.* 2014; Barelli *et al.* 2016). Considering entomopathogenicity, the genus *Metarhizium* could be categorized as narrow host range, intermediate, and broad host range insect pathogens. Examples of species with a narrow host range that infects insects in only one order are *Metarhizium* *rileyi*, which infects species only in the order Lepidoptera (Fronza *et al.* 2017), *M. album* that infects species only in the order Homoptera (Rombach *et al.* 1987) and *M. acridum*, that infects species only in the order Orthoptera (Stone and Bidochka 2020). *M.* *guizhouense* and *M.* *majus* could be categorized as intermediate host range, infecting insects from a few orders such as Coleoptera and Lepidoptera (Hu *et al.* 2014; Stone and Bidochka 2020). Conversely, *M.* *anisopliae*, *M.* *robertsii*, and *M.* *bruneum* are examples of fungi with a broad host range that infect ticks, mites, and several orders of insects (Hu *et al.* 2014; Stone and Bidochka 2020). Brazil has a long tradition of using microbial products for the biocontrol of arthropods, and the spraying of *M. anisopliae* s.s. as a bioproduct in sugarcane against spittlebugs (Hemiptera: Cercopidae) represents one of the most extensive microbial control programes worldwide (Iwanicki *et al.* 2019; Mascarin *et al.* 2018).

The practical functions of endophytic microbes might include obtaining and transferring nutrients from soil to plants, modulating plant development, increasing the stress tolerance of plants, activating plant defense against invertebrate pests, suppressing virulence in plant pathogens, and suppressing the growth of competitor plant species (Hu and Bidochka 2019; White *et al.* 2019). Promising results show that several Brazilian *Metarhizium* spp. isolates function as a plant biostimulant and induce defense against pest insects and mites and diseases in tomato (Siqueira *et al.* 2020), maize (Lira *et al.* 2020), strawberry (Canassa *et al.* 2019a, 2019b), and common bean plants (Canassa *et al.* 2019c).

Soil is a well-known reservoir of entomopathogenic fungi (Vega *et al.* 2012, 2018), and the diversity of indigenous *Metarhizium* spp. from soils in five Brazilian biomes, which represent approximately 93% of the Brazilian land area, was recently characterized (Riguetti Zanardo Botelho *et al.* 2019) . *Metarhizium* *robertsii sensu stricto* (s.s.) was the most abundant species, followed by *M.* *humberi* sp. nov., *M. anisopliae* s.s., *M. pingshaense* (s.s.), and two other lineages that lie beyond currently recognized species. *Metarhizium* *humberi* stands out from the other species by the highest haplotype and nucleotide diversities among isolates (38 isolates with 15 different haplotypes based on the nuclear intergenic region MzlGS3). This new species was recently assigned as a new member of the PARB clade [composed of strains belonging to *M. pingshaense* (s.s.), *M. anisopliae* (s.s.), *M. robertsii* (s.s.), and *M. brunneum* (s.s.)] within the *M. anisopliae* complex, occurring in soil from predominantly conserved areas of native savanna in Central Brazil’s Cerrado biome, and has also been isolated from coleopteran, hemipteran and lepidopteran insects in Brazil and Mexico (Luz *et al.* 2019). *Metarhizium* *humberi* proved to be highly virulent against the two-spotted spider mite *Tetranychus urticae* (Castro *et al*. 2018), and has broad suitability for use as a biological control agent against pests of medical, veterinary, and agricultural importance. Concerning the potential to establish plant associations, a recent study showed that *M. humberi* (s.s.), *M. robertsii* (s.s.), and *M. anisopliae* (s.s.) improved the leaf area, plant height, root length and dry weight of corn plants compared to un-inoculated corn plants (Lira *et al.* 2020). Corn plants treated with any of the three species of *Metarhizium* significantly reduced the survival time of the caterpillar *Spodoptera frugiperda* that fed on treated plants (Lira *et al.* 2020). Furthermore, *M. humberi* is under consideration for registration as a bioproduct in Brazil.

Genome sequences of 17 *Metarhizium* strains are available to date, including representatives of the PARB clade (*M. anisopliae*, *M. robertsii*, and *M. brunneum*), MGT clade (*M. majus* and *M. guizhouense*), and three other species: *M. acridum*, *M. rileyi*, and *M. album*. In the last decade, genome sequencing technologies have produced breakthroughs in several fields and improved understanding of the mechanisms of interactions between insects, plants, and entomopathogens (Wang *et al.* 2016). All this knowledge will potentiate bioproduct' cost-effective applications for pest control in the field (Wang and Wang 2017). In this study, our main objective was to characterize the genome of the newly described species *M. humberi* (strain ESALQ1638), recovered from soil in Brazil, which has excellent potential as a new biopesticide product. We hypothesize that some elements of the *M. humberi* genome, such as the diversity of specific enzymes and composition of secondary metabolites (SMs), differ from the closely related species and broad host range *M. anisopliae* and *M. robertsii* whereas others elements, such as enzymes associated to nutrient acquisition and niche adaptation like proteases and carbohydrate-related enzymes are present in similar number in *M. anisopliae* and *M. robertsii* genome. We used a multifaceted bioinformatics approach and identified genes with putative functions based on existing *Metarhizium* spp. and outgroups emphasizing similarities and differences between diverse lifestyles (pathogenic to insects and mites, saprophytic and endophytic), and used phylogenomics to show its evolutionary relationships.

## Materials and methods

### Origin and culture of fungal strain ESALQ1638

A new Brazilian species named *M.* *humberi* (strain ESALQ1638) was selected for whole-genome sequencing after ongoing studies by our team (Laboratory of Pathology and Microbial Control of Insects—LPCMI, of the Escola Superior de Agricultura “Luiz de Queiroz” at University of São Paulo, ESALQ/USP, in Piracicaba city, state of Sao Paulo, Brazil), which indicated its probiotic effect in plants and pathogenicity against important pests (Siqueira *et al.* 2020 and unpublished results). Originally, *M. humberi* strain ESALQ1638 was isolated from soil of native vegetation of a Cerrado biome, a savanna-like grassland in Rio Verde city (17°29'49,3''S; 51°13'40,7''W; 858 m altitude), state of Goiás (GO) Brazil. The access of *M. humberi* strain ESALQ1638 is registered at the Brazilian System for the Management of Genetic Heritage and Associated Traditional Knowledge—SisGen under the code RAC856E. A single spore culture of ESALQ1638 was prepared on potato dextrose agar (PDA) (Difco^®^). Then, it was grown on sabouraud dextrose and yeast (SDY)/4 liquid medium (2.5 g l^−1^ peptone, 10 g l^−1^ dextrose, 2.5 g l^−1^ yeast extract) at 25°C for 5 days on a shaker (125 rpm) before mycelium was vacuum filtered and harvested for storage at −80°C. Stock cultures of this strain are maintained in the Collection of Entomopathogenic Microorganisms of LPCMI at ESALQ/USP, Brazil.

### Genome sequencing, de novo assembly, gene prediction, and annotation

Genomic DNA was extracted from mycelium, obtained as described in the previous section, using a phenol and chloroform protocol, as described in Rezende *et al.* (2015). The DNA quality was assessed through fluorometry (QuBit) and UV absorption (Nanodrop). Sufficient genomic DNA was then prepared for paired-end and mate-pair (3 and 5 kb) sequencing at the Center of Functional Genomics (ESALQ/USP, Brazil) using Illumina HiSeq 2500, generating more than 564 million Illumina paired-end reads (coverage of >1.000-fold) with a 101 bp length. Graphical assessment of raw read data quality was conducted using the software FASTQC ver.0.11.5 (Andrews 2010).

Raw read sequences were trimmed via Trimmomatic (ver. 0.36) (Bolger *et al.* 2014) for paired-end sequences and NxTrim (ver. 0.4.2) (O’Connell *et al.* 2015) for both mate pairs. Assemblies and scaffolding were performed using four *de novo* assemblers: CLC Genomics Workbench 7.5 (Qiagen), SOAPdenovo2 (ver. 2.0.4) (Luo *et al.* 2012), SPAdes (ver. 3.11.0) (Bankevich *et al.* 2012), and IDBA-UD (ver. 1.1.3) (Peng *et al.* 2012) using a broad range of k-mer values (from 21 up to 100). The single best k-mer value for de novo assembler SOAPdenovo2 was predicted using KmerGenie (ver. 1.7044) (Chikhi and Medvedev 2014). In general, the default for all computer programs was selected. Scaffolds smaller than 200 bp were removed using CLC Genomics Workbench 7.5 (Qiagen) before quality metrics of all assemblers were calculated using the QUAST assessment tool (ver. 4.5) (Gurevich *et al.* 2013).

Before gene prediction, the repeats in the scaffolds were masked using RepeatMasker (ver. 4.0.7) (http://repeatmasker.org) with CrossMatch (http://www.phrap.org/phredphrapconsed.html). Gene prediction was performed with Augustus (ver. 3.03) (Stanke *et al.* 2004), using default settings and *Fusarium graminearum* as a training set. Protein sequences were blasted against the NCBI nr database and annotated using Blast2GO^®^ (ver. 4.1.9) (Gotz *et al.* 2008), applying the Annex augmentation. InterProScan was performed with Blast2GO^®^ retrieving domain/motif information to the sequences and merged with existing GO terms.

Protein sequences were clustered into orthogroups (orthologues and paralogues) using Orthofinder2 (Emms and Kelly 2015, 2019). The Orthofinder2 log files, which include the exact command used to run the analysis, and all results can be accessed on the GitLab repository (https://gitlab.nibio.no/simeon/iwanicki_et_al_21). RepeatMasker and Augustus were used in all species to find repetitive sequences and predict the genes in the other *Metarhizium* genomes, respectively, to use identical gene prediction parameters in all genomes before comparison. Venn diagram was made in the web-based tool: InteractiVenn (Heberle *et al.* 2015). Noncoding RNAs were identified using default settings with both tRNAscan-SE (ver. 2.0) (Lowe and Eddy 1997) and Infernal (ver. 1.1.2) (Nawrocki and Eddy 2013).

### Comparative genomics

The genome of the *M. humberi* strain ESALQ1638 was compared to ten previously sequenced genomes of the *Metarhizium* genus (*M. anisopliae* BRIP53293, *M. anisopliae* ARSEF549, *M. anisopliae* ESALQE6, *M. robertsii* ARSEF23, *M. brunneum* ARSEF3297, *M. guizhouense* ARSEF977, *M. majus* ARSEF297, *M. acridum* CQMa102, *M. album* ARSEF1941, *M. rileyi* RCEF4871). All *Metarhizium* genomes were obtained from the NCBI genome database in June 2017 (http://www.ncbi.nlm.nih.gov/genome/), and detailed information about the geographic origin and host/substrate from each *Metarhizium* strain can be accessed at the supplementary material (Additional file 1, Table S2).

Pairwise comparison of the genome of ESALQ1638 to other published *Metarhizium* genomes was performed using the average nucleotide identity (ANI) calculator (Rodriguez-R and Konstantinidis 2014), and the genome-to-genome-distance calculator GGDC 2 (http://ggdc.dsmz.de/home.php) was used to predict digital DNA–DNA hybridization (dDDH) values (Meier-Kolthoff *et al.* 2013). Whole-genome alignments between the genome assemblies of all *Metarhizium* considering ESALQ1638 as a reference were performed with NUCmer (NUCleotide MUMmer) (version 3.1) and default parameters. Dot plots were generated using the GenomicRanges and tidyverse packages in R (Lawrence *et al.* 2013; Wickham *et al.* 2019). In short, the nucmer results of all contig to contig alignments were parsed into tabular information, short-length alignments (<1000 bp) were filtered out, and remaining alignments were plotted. R script is provided as an additional file (Additional file 2) and deposited on the GitLab repository (https://gitlab.nibio.no/simeon/iwanicki_et_al_21/-/tree/master/Contig_sorting).

Transposable elements were analyzed using the program RepeatMasker v4.0.7 using CrossMatch (http://www.phrap.org/phredphrap/general.html) and repeat-induced point mutations (RIPs) were estimated with RIPer (www.theripper.hawk.rocks).

### Functional annotation and analysis of gene family evolution

Functional annotation of *M. humberi* ESALQ1638 was conducted using Blast2GO^®^ (Al-Shahrour *et al.* 2004) and InterProScan. InterProScan was used to determine putative functional annotation of the predicted protein-coding genes in eight *Metarhizium* species and four outgroups (*Beauveria bassiana* ARSEF2860, *Aspergillus niger* SH-2, *Fusarium verticillioides* 7600, and *Trichoderma harzianum* T6776). These functional annotations were used to create GO term databases for each of eleven genomes from eight *Metarhizium* species to determine the function of overrepresented genes belonging to rapidly evolving gene families according to CAFE5 (Mendes *et al.* 2020). The full analysis of overrepresented GO terms in fastly evolving gene families described in this paragraph is reproducibly documented in an R-Markdown file (“cafe5_GOstats_parser.Rmd”) that can be accessed from the public GitLab repository (https://gitlab.nibio.no/simeon/iwanicki_et_al_21). In short, Orthofinder2 was used on eleven *Metharhizium* genomes; the Orthofinder2 species tree with additional time-calibration was used as the ultrametric tree for CAFE5 and the orthogroups were used as the gene families for CAFE5. Gene lists comprising all members of fastly evolving gene families were extracted for each of the eleven genomes. These gene lists were then used to test for overrepresentation of GO terms in the genome-specific GO term databases created on the basis Blast2GO functional annotation. Overrepresentation was determined by hypergeometric testing with additional conditioning on GO term hierarchical structure as implemented in the GOstats R package (*P* < 0.05) (Falcon and Gentleman 2007). To classify serine peptidases' (SP) families, the proteome of *M. humberi* ESALQ1638, *M. robertsii* ARSEF23, and *M. anisopliae* isolates BRIP53 were identified by Blastp searching against the MEROPS peptidase database Release 11 (http://merops.sanger.ac.uk/) with a cutoff *E*-value < 0.001.

### Identification of secondary metabolites

Genes annotated as polyketide synthases (PKSs), nonribosomal peptide synthetases (NRPSs), hybrid NPKS-PKS in *M. humberi* ESALQ1638, and eight other *Metarhizium* genomes were determined using Blast2GO^®^ (Al-Shahrour *et al.* 2004) and a review of SMs that have been isolated from nine sequenced *Metarhizium* species (Donzelli and Krasnoff 2016). Donzelli and Krasnoff (2016) classified families of secondary metabolism genes from nine *Metarhizium* species based on orthology, phylogenetic analysis, and conservation of gene organization.

### Secretome prediction

Many bioinformatics tools have been developed for predicting subcellular locations of proteins and secretion to the extracellular space (Caccia *et al.* 2013). However, a combination of these tools is required for reliable secretome annotation. Here, the secreted proteins of *M. humberi* ESALQ1638, *M. robertsii* ARSEF23, *M. anisopliae* BRIP53293 and *M. rileyi* RCEF4871 were predicted using a well-evaluated protocol adapted from Staats *et al.* (2014) and Brown *et al.* (2012), which includes the bioinformatics tools: SignalP v5.0 (http://www.cbs.dtu.dk/services/SignalP/index.php) and TargetP v1.1 (Loc = S) (http://www.cbs.dtu.dk/services/TargetP/) for signal peptide prediction, TMHMM v2.0 (http://www.cbs.dtu.dk/services/TMHMM) for identifying proteins having transmembrane domains (Emanuelsson *et al.* 2007), WolF PSort (https://wolfpsort.hgc.jp/) (Horton *et al.* 2007) for subcellular location prediction (extracellular score > 17) and PROSITE-Scan (https://prosite.expasy.org/scanprosite/) for identifying endoplasmic reticulum (ER) target protein (Prosite = PS00014) (Castro *et al.* 2006; Xiang 2010). Thus, the secretomes defined here are proteins from the annotated genome of *M. humberi*, *M. robertsii* ARSEF23, *M. anisopliae* BRIP53293, and *M. rileyi* RCEF4871 predicted as having a signal peptide at their N-terminus by SignalP and TargetP, no transmembrane domain or a transmembrane domain located in the first 60 amino acids in the N-terminal region as predicted by TMHMM (Brown *et al.* 2012; Staats *et al.* 2014) and with a subcellular location predicted as extracellular by WolfPsort, but not having an ER targeting signal (PS00014). In addition, we performed a functional analysis of predicted secreted proteins by assigning them to respective gene ontology (GO) groups, protein family domains (Pfam) for *M. humberi*, Carbohydrate-Active Enzymes (CAZymes), and secreted enzyme [Enzyme Commission (EC) numbers] in *M. humberi*, *M. robertsii* ARSEF23, *M. anisopliae* BRIP53293, and *M. rileyi* RCEF4871. The web server dbCAN2 (Zhang *et al.* 2018) was used to annotate carbohydrate-active enzymes (CAZy) in *Metarhizium* proteome with parameters were as follows: for HMMER, *E*-value < 1e-^15^ and coverage > 0.35.

### Phylogenetic relationship of *Metarhizium* species from the PARB clade in relation to other ascomycete fungi

Orthologs of 11 *Metarhizium* genomes (*M. acridum* CQMa1023; *M. album* ARSEF1941; *M. anisopliae* ARSEF5493, BRIP53293 and ESALQE6; *M. brunneum* ARSEF32973; *M. guizhouense* ARSEF9773; *M. humberi* ESALQ1638; *M. majus* ARSEF2973; *M. rileyi* RCEF48713; *M. robertsii* ARSEF233) and four outgroups (*A. niger* SH23, *B. bassiana* ARSEEF28603, *F.* *verticillioides* 7600 and *T. harzianum* T67763) were determined using the software OrthoFinder (version 2.5.2) with default settings (Emms and Kelly 2019). All 3131 orthogroups with exactly one member in each genome (one-to-one orthogroups) were selected for phylogeny analysis. The protein sequences from those genes and concatenated in identical order to form a single long protein sequence for each species. The alignment and phylogenetic tree were generated in R using, among others, the DECIPHER, ape, phangorn packages. The R script, including versions used for R and all packages used to generate the tree is available at the supplementary material (Additional file 3) and GitLab repository associated with this article. In short, a maximum-likelihood phylogeny was calculated and fitted with the LG substitution model (Le and Gascuel 2008). The estimations for gamma rate and evolutionarily invariable sites were optimized with the fit of the LG substitution model. Bootstrap analysis was performed to estimate the robustness of the generated tree (1000 bootstraps). This was accomplished for all analyzed genomes and a subselection containing only the *Metarhizium* genomes for improved resolution. In the analysis of all genomes, *A. niger* was selected as the outgroup to root the tree on. The tree was drawn to scale, with branch lengths measured in the number of substitutions per site.

### Genes involved in pathogen–host interactions

The pathogen–host interactions (PHI) database (version 3.2, http://www. phi-base.org/) was used to search for orthologs proteins in *M. humberi* ESALQ1638, *M. anisopliae* BRIP53293 and *M. robertsii* ARSEF23 associated to virulence to arthropods and plant interaction. The matches were filtered using *e*-value ≤10^−5,^ and only proteins that shared over 50.0% identity with *Metarhizium* predicted proteins and associated with “increased virulence” toward the invertebrate host and “effectors” as phenotype characteristics were considered.

## Results

### *Metarhizium humberi* genome sequences and assembly

The genome of the *M. humberi* isolate ESALQ1638 was sequenced using Illumina paired-end and mate-pair libraries, and contigs and scaffolds were assembled using four de novo assemblers. The genome size was similar among the assemblers used, varying from 38.07 to 39.30 Mb. However, there were significant differences between other metrics obtained from assemblers, such as numbers of contigs, scaffolds, and gaps. The assembly results provided by SOAPdenovo 2 showed the lowest number (*n* = 291) and the longest scaffolds (7.15 Mb); however, the number of gaps was very high (*n* = 1.177.903). The best assembler was IDBA‐UD, which generated a reduced number of gaps (*n* = 11124), an acceptable number of scaffolds (*n* = 1072), an N50 length of 1.4 Mb, and a G + C content of 49.8%. Assembly results from IDBA-UD were then processed with GapFiller (ver. 1.10) (Boetzer and Pirovano 2012) that reduced scaffold gaps by 55.8%. The comparative results from genome assembly in different programs are provided in Supplementary table S1 (Additional file 1). The assembly statistics and general features of *M. humberi* ESALQ1638 are shown in Table  1.

**Table 1:** Summary of genome assembly and annotation features of *M. humberi* ESALQ1638

Features	*M. humberi* ESALQ1638
Size (Mb)	38.33
Coverage (fold)	>1000×
Number of contigs	1081
Number of scaffolds^a^	1072
Number of large scaffolds (>100 kb)	44
Number of large scaffolds (>1 Mb)	12
Longest scaffold (Mb)	3.87
N50 scaffold length (Mb)	1.40
L50 scaffold count	9
G + C content (%)	49.84
N's per 100 kbp	16.14
Number of predicted genes	10633
Average no of genes per Mb	360.45
Mean gene length (base pairs)	1732.89
Exons per gene	2.80
tRNA	141

a ≥ 200 nucleotides.

### Whole-genome synteny comparisons, pairwise ANIs, and phylogenetic analysis between *Metarhizium* species

**Figure 1:**
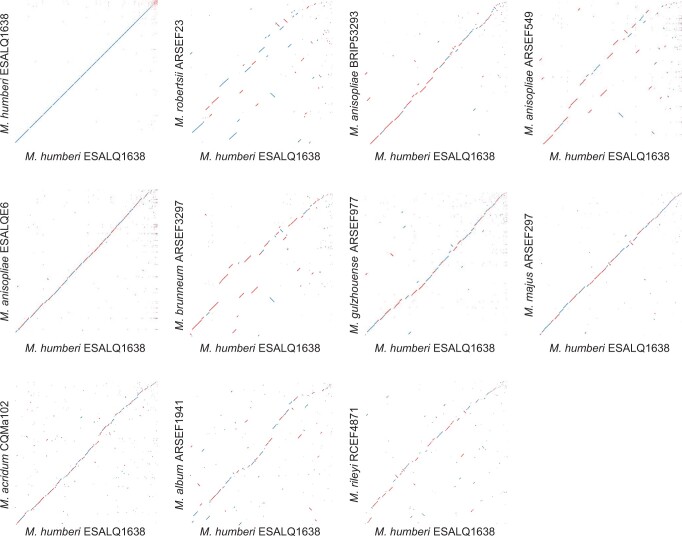
Dot-plots representing whole-genome comparison between *M. humberi* ESALQ1638 and 10 other *Metarhizium* strains. The comparison was performed using NUCmer 3.1 for each pair of genomes. Scaffolds of *M. humberi* ESALQ1638 are displayed by decreasing size along the *x*-axis, matching scaffolds of the compared genome are shown on the *y*-axis. Homologous regions are plotted as diagonal lines with dots at the starting and endpoints. Color coding indicates an aligned strand, with blue representing the main strand and red showing the reverse strand.

We generated dot plots for each pair of genomes to identify synteny between *M. humberi* ESALQ1638 and 10 *Metarhizium* isolates from eight *Metarhizium* species. We found high levels of sequence homology between the genomes of *M. humberi* ESALQ1638 and the broad host range fungus *M. robertsii* ARSEF23, followed by *M. anisopliae* BRIP53293, *M. anisopliae* ARSEF549, and *M. anisopliae* ESALQE6 (Figure  1). In contrast, lower sequence homology was found between *M. humberi* ESALQ1638 and the narrow host range fungus *M. rileyi* RCEF4871 (Figure  1).

Corroborating the results from synteny dot-plot analyses, a higher pairwise ANI was found between *M. humberi* ESALQ1638 and *M. anisopliae* BRIP53293 (97.0%) and between *M. humberi* ESALQ1638 and *M. robertsii* ARSEF23 (96.8%). Conversely, a lower pairwise ANI was found between *M. humberi* ESALQ1638 and *M. rileyi* RCEF4871 (80.6%) (Table  2). The number of coding sequences (CDS) and genome size was similar between broad host range species, *M. robertsii* (41 Mb, 11,688 CDS), *M. anisopliae* (38 Mb, 10,891–11,415 CDS), and *M. brunneum* (37 Mb, 10,689 CDS) (Table  2).

**Table 2:** Pairwise ANI between the *M. humberi* ESALQ1638 and other *Metarhizium* species, with their respective genome sizes and numbers of coding sequences (CDSs)

Species isolate	Genome size (Mb)	Number of CDSs	Pairwise ANI (%)
			ESALQ1638
*M. humberi* ESALQ1638	38.33	10,633	
*M. anisopliae* BRIP53293	38.67	11,415	96.95
*M. robertsii* ARSEF23	41.66	11,688	96.84
*M. anisopliae* ARSEF549	38.50	10,891	96.80
*M. anisopliae* ESALQE6	38.47	10,958	96.78
*M. brunneum* ARSEF3297	37.07	10,689	95.57
*M. guizhouense* ARSEF977	43.47	11,787	94.53
*M. majus* ARSEF297	42.06	11,535	94.30
*M. acridum* CQMa102	38.05	9,974	88.33
*M. album* ARSEF1941	30.45	8,472	82.39
*M. rileyi* RCEF4871	32.01	8,764	80.56

**Figure 2:**
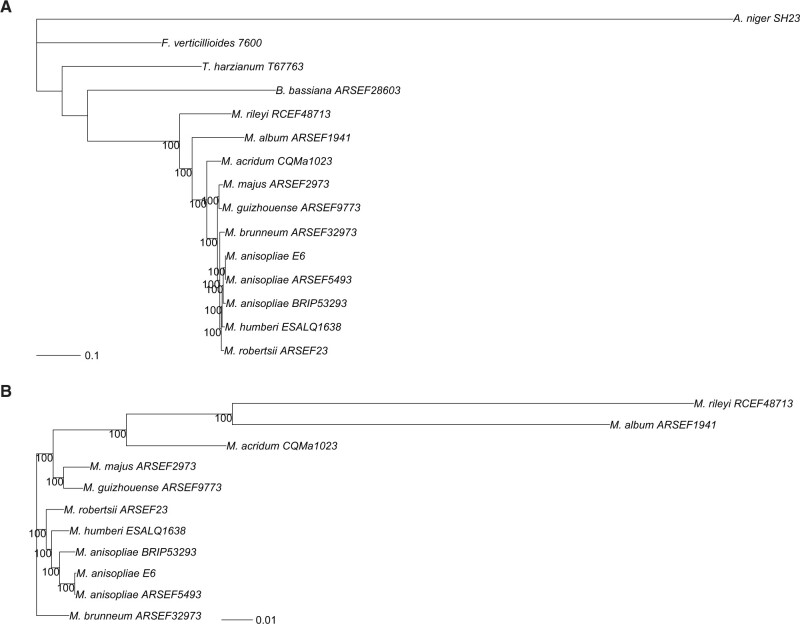
Maximum likelihood tree using concatenated protein sequences of 3131 orthologous genes with exactly one ortholog in the genomes of *A. niger* SH23; *B. bassiana* ARSEEF28603; *F. verticillioides* 7600; *M. acridum* CQMa1023; *M. album* ARSEF1941; *M. anisopliae* ARSEF5493, BRIP53293 and ESALQE6; *M. brunneum* ARSEF32973; *M. guizhouense* ARSEF9773; *M. humberi* ESALQ1638; *M. majus* ARSEF2973; *M. rileyi* RCEF48713; *M. robertsii* ARSEF233; and *T. harzianum* T67763. The evolutionary history was inferred by using the maximum likelihood method with an LG model. The tree is drawn to scale, with branch lengths measured in the number of substitutions per position.

Next, we examined the evolutionary relationships in *M. humberi* ESALQ1638 and the closely related species *M. anisopliae* and *M. robertsii* based on a phylogeny derived from 3131 orthologous protein-coding genes represented by one orthologue in each genome. The tree supports the results previously shown and resolved *M. humberi* ESALQ1638 as a sister group of *M. anisopliae* and closer to *M. robertsii* than to *M. brunneum* (Figure  2).

### Noncoding RNAs, transposable elements, and repetitive DNA content

Approximately 2.0% of the *M. humberi* ESALQ1638 genome is DNA transposons, and 15.0% retroelements [Supplementary Table S3 (Additional file 4)]. Among the different retroelements, the long terminal repeats (LTRs) and long interspersed nuclear elements (LINEs) were most abundant, representing 12.05 and 3.0% of the genome, respectively. The *M. humberi* ESALQ1638 genome was less repetitive than the genomes of *M. robertsii* ARSEF23 and *M. anisopliae* BRIP53293, ARSEF549, and ESALQE6 due to the lower content of LTRs and LINEs [Supplementary Tables S3 and S4 (Additional files 4 and 5)]. A total of 217 putative retroelements and 62 DNA transposons were identified in the *M. humberi* genome [Supplementary Table S3 (Additional file 4)], approximately 100 fewer than were found in *M. robertsii* ARSEF23 and *M. anisopliae* isolates, with between 300 and 330 putative retroelements and 119 to 179 DNA transposons, respectively. However, the number of transposable elements found in *M. humberi* ESALQ1638 was similar to those found in *M. brunneum* ARSEF3297: 194 putative retroelements and 66 DNA transposons [Supplementary Table S4 (Additional file 5)]. In the *genome* of the fungus *M. rileyi* RCEF4871, we also identified similar numbers of putative retroelements (*n* = 202) and DNA transposons (*n* = 91) compared to the *M. humberi* ESALQ1638 genome.

In the *M. humberi* ESALQ1638 genome, 141 tRNAs were identified by tRNAscan-SE, out of which six were found to be pseudogenes. The number of tRNAs occurring in other *Metarhizium* species is provided in the supplementary material (Additional file 8).

A repeated-induced point mutation (RIP) is a genomic defense mechanism exclusively found in fungi that mutates duplicated sequences to avoid transposon replication (Gladyshev 2017). We found that *M. humberi* ESALQ1638 has more RIP than *M. robertsii* ARSEF23 and *M. anisopliae* BRIP53293, affecting 5.8% of the *M. humberi* ESALQ1638 total genomic TE content (Table  3). While 51 large RIP-affected regions (LRARs) were identified in the *M. humberi* ESALQ1638 genome, only one and three regions were found in the *M. robertsii* ARSEF23 and *M. anisopliae* BRIP53293 genomes, respectively.

**Table 3:** Repeat-induced point mutation signatures in the *M. humberi* ESALQ1638, *M. robertsii* ARSEF23, and *M. anisopliae* BRIP53293 genomes

Species	*M. humberi*	*M. robertsii*	*M. anisopliae*
Count of genomic windows investigated	77,187	83,313	77,344
Number of RIP affected windows	4,534	218	309
RIP affected genomic proportion (%)	5.84	0.26	0.4
Count of LRARs	51	1	3
Average Size of LRARs (bp)	7,737.41	5,500	4,666.67
Average GC content of LRARs (%)	20.03	33.22	25.88
Genomic proportion of LRARs (bp)	394,608	5,500	14,000
Product value for LRARs	1.47	1.61	1.69
Substrate value for LRARs	0.3	0.6	0.6
Composite value for LRARs	1.17	1.01	1.09

LRAR, large RIP affected regions; RIP, repeat-induced point mutation.

### Transcription factors

To identify the number of transcription factors, we computed InterPro categories assigned to transcriptional regulation in the *M. humberi* ESALQ1638 genome and eight other *Metarhizium* spp. [Supplementary Table S5 (Additional file 6)]. There was a similar number of transcription factors in *M. humberi* ESALQ1638, *M. robertsii* ARSEF23, *M. anisopliae* ESALQE6, ARSEF549 and BRIP53293, *M. brunneum* ARSEF3297, *M. guizhouense* ARSEF977, and *M. majus* ARSEF297 (Supplementary Table S5), of which Zn(2)-C6 fungal-type DNA-binding domain (IPR001138) and generic fungal transcription factors (IPR007219) were the most abundant with 167 in all *Metarhizium* species and 162 in the *M. humberi* ESALQ1638 genome. In general, *M. album* ARSEF1941 and *M. rileyi* RCEF4871 had the lowest number of transcription factors [Supplementary Table S5 (Additional file 6)].

### Gene prediction and predicted protein orthology

**Figure 3:**
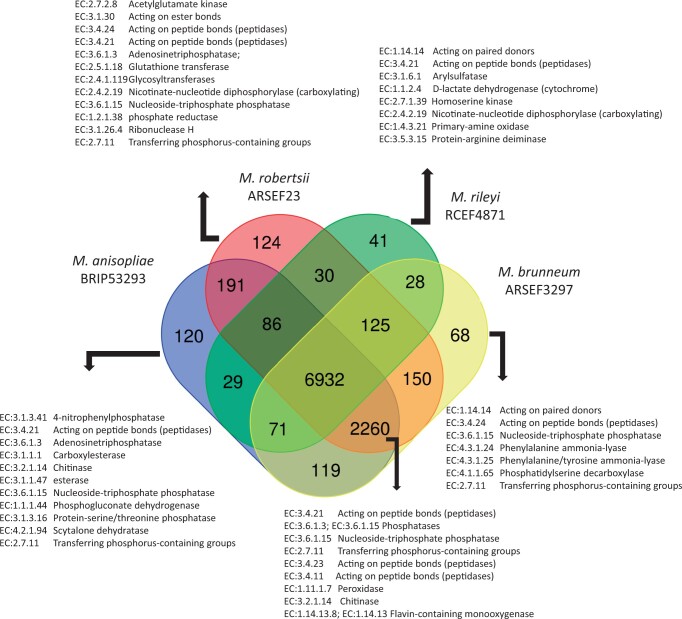
Venn diagram comparing the orthologous of *M. humberi* ESALQ1638 shared between *M. rileyi* RCEF48713, *M. robertsii* ARSEF23, *M. anisopliae* BRIP53293, and *M. brunneum* ARSEF3297 and enzymes annotated to shared orthologous (EC: enzyme code).

A total of 10,633 protein-encoding genes were predicted in the *M. humberi* ESALQ1638 genome assembly, compared with 11,415 in *M. anisopliae* BRIP 53,293 and 11,688 in *M. robertsii* ARSEF23 (Table  2). The orthology analyses revealed that a total of 9192 proteins in the *M. humberi* ESALQ1638 genome were orthologs in common in *M. robertsii* ARSEF23, *M. anisopliae* BRIP53, and *M. brunneum* ARSEF3297 while only 6932 proteins were in common in *M. rileyi* RCEF4871 genome (Figure  3). However, 124, 120, 68, and 41 proteins in *M. humberi* ESALQ1638 were orthologs only in *M. robertsii* ARSEF23, *M. anisopliae* BRIP53293, *M. brunneum* ARSEF3297, and *M. rileyi* RCEF4871, respectively (Figure  3 and Additional file 9). These proteins might have orthologs in other *Metarhizium* species. Among the 124 genes shared only between *M. humberi* ESALQ1638 and *M. robertsii* ARSEF23, we highlighted the enzymes: kinases, peptidases, glycosyltransferase, ribonuclease, proteases and phosphatases major facilitator substrate transporter, potassium transporters, nitroreductases, PKSs, and GABA permease (Figure  3 and Additional file 9). Conversely, among the 120 orthologous proteins shared only with *M. anisopliae* BRIP53293 are enzymes, such as chitinase, phosphatase, esterases, peptidases, and cytochrome P450, sugar transporter, gibberellin oxidase, lovastatin nonaketide synthase, and VOC family (Additional file 9). Conversely, within the orthologs proteins shared between *M. anisopliae* BRIP53293, *M. robertsii* ARSEF23, and *M. brunneum* ARSEF3297 but not with *M. rileyi* RCEF4871 are enzymes such as peptidases, representing the largest group of enzymes (*n* = 66), followed by phosphatases (*n* = 44), peroxidases (*n* = 7) and chitinases (*n* = 7) (Figure  3 and Additional file 9). In addition are included in this group homologs to well-studied insect cuticle and plant binding adhesin *Mad1* (MHUMG1_05216) and *Mad2* (MHUMG1_05182), homologs to ammonium transporters *Mep2* (MHUMG1_01898), *MepC* (MHUMG1_06521), urease (MHUMG1_00106), subtilisin-serine protease *Pr1* (MHUMG1_2023) (Moonjely and Bidochka 2019), homologs to raffinose transporter *Mrt* (MHUMG1_01550) (Fang and St. Leger 2010) and Membrane anchor *Opy2* (Guo *et al.* 2017) (MHUMG1_02796). The lowest percentage of orthologs was found in *M. album* ARSEF1941 (67.4%), followed by *M. rileyi* RCEF 4871 (69.0%). The complete list of orthologous proteins shared between *M. humberi* and others can be found in the supplementary material (Additional file 9).

InterProScan within Blast2GO was used to retrieve the family/domain information for all *Metarhizium* protein sets. We found that ESALQ1638 has 5186 protein families and 9039 protein domains, compared to 5226 families and 9184 domains in *M. robertsii* ARSEF23, 4178 families, and 7110 domains in *M. album* ARSEF1941 [Supplementary Table S6 (Additional file 7)].

### Functional annotation

**Figure 4:**
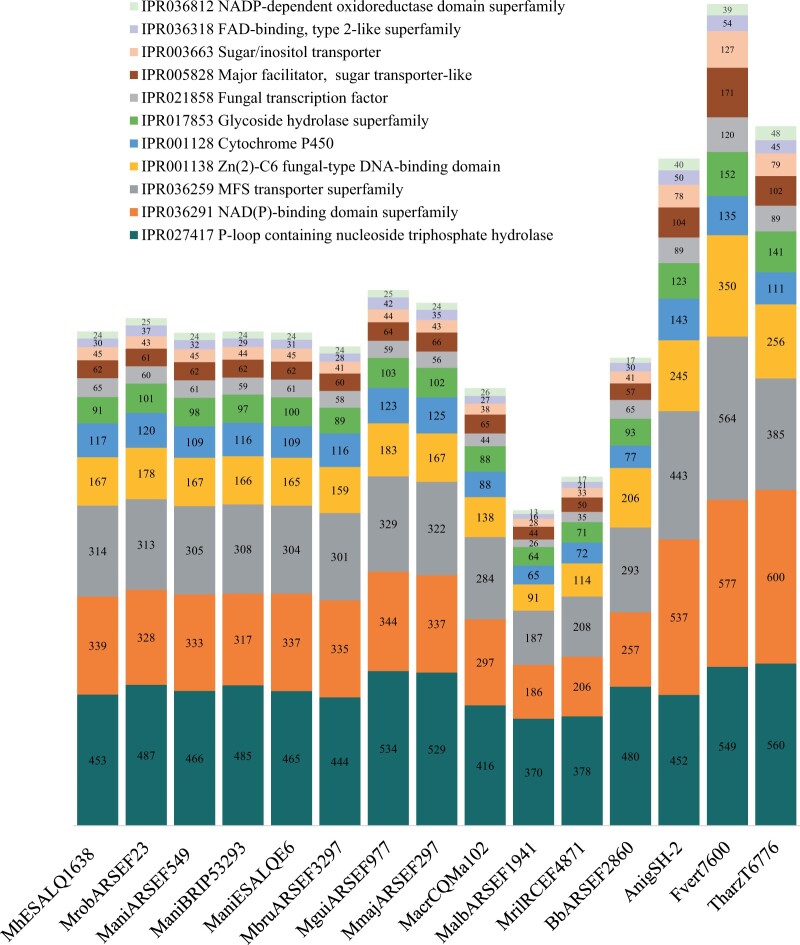
Interpro categories and number of predicted protein-coding genes from each category found in the genome of *M. humberi* ESALQ1638, eight *Metarhizium* species, *B. bassiana* ARSEF2860, *A. niger* SH-2, *F. verticillioides* 7600, and *T. harzianum* T6776. Mrob (*M. robertsii* ARSEF23); ManiA (*M. anisopliae* ARSEF549); ManiB (*M. anisopliae* BRIP53293); ManiE (*M. anisopliae* ESALQE6); Mbru (*M. brunneum* ARSEF3297); Mgui (*M. guizhouense* ARSEF977); Mmaj (*M. majus* ARSEF297); Macr (*M. acridum* CQMa102); Malb (*M. album* ARSEF1941); and Mril (*M. rileyi* RCEF4871).

**Figure 5:**
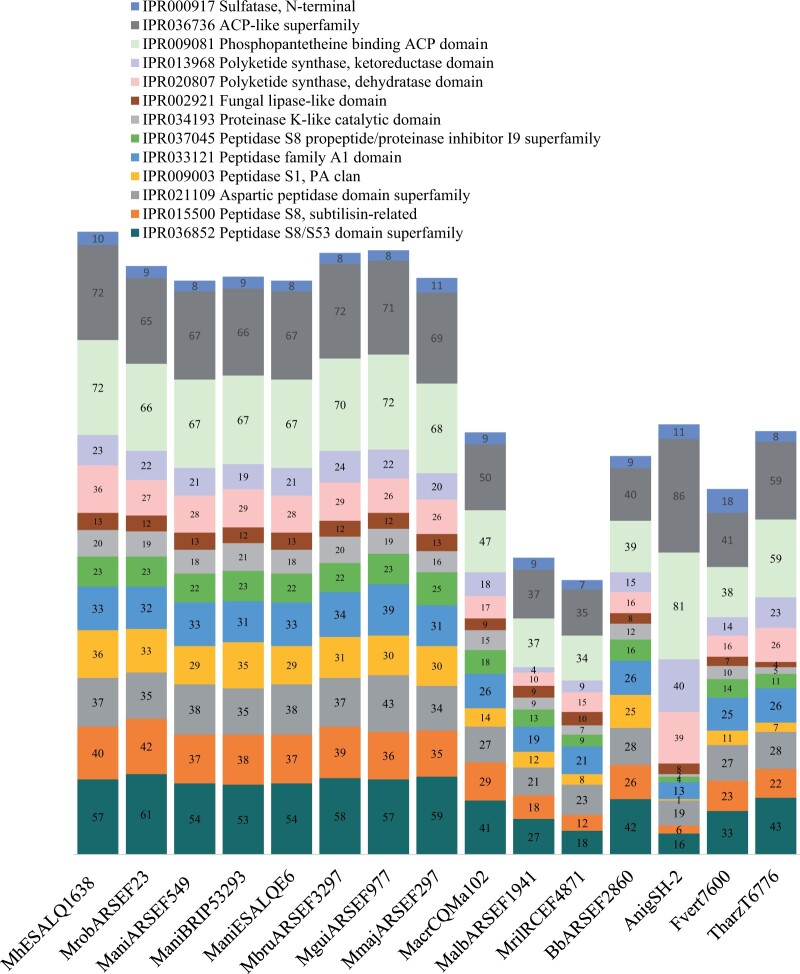
Interpro categories and number of predicted protein-coding genes from each category found in the genome of *M. humberi* ESALQ1638, eight *Metarhizium* species, *B. bassiana* ARSEF2860, *A. niger* SH-2, *F. verticillioides* 7600, and *T. harzianum* T6776. Mrob (*M. robertsii* ARSEF23); ManiA (*M. anisopliae* ARSEF549); ManiB (*M. anisopliae* BRIP53293); ManiE (*M. anisopliae* ESALQE6); Mbru (*M. brunneum* ARSEF3297); Mgui (*M. guizhouense* ARSEF977); Mmaj (*M. majus* ARSEF297); Macr (*M. acridum* CQMa102); Malb (*M. album* ARSEF1941); and Mril (*M. rileyi* RCEF4871).

InterProScan was used to determine putative functional annotation of the predicted protein-coding genes in eight *Metarhizium* species and four outgroups (*B.* *bassiana* ARSEF2860 (an entomopathogenic fungus), *A.* *niger* SH-2 (an opportunistic fungus), *F.* *verticillioides* 7600 (a plant pathogenic fungus), and *T.* *harzianum* T6776 (a mycoparasite that is also used as a fungicide) (Additional file 10). To illustrate the main differences between those fungi, we selected InterPro categories common to most fungi, such as primary metabolism and general fungal life cycle (Figure  4). In a second investigation, we focused on InterPro categories associated with specific enzymes involved in degrading substrates, acquiring nutrients, and SMs, such as proteases, peptidases, lipases, and PKSs and sulfatases (Figure  5).

In general, the numbers of protein-coding genes related to typical fungal lifestyle were similar between *M. humberi* ESALQ1638, *M. robertsii* ARSEF23, the three *M. anisopliae* isolates ESALQE6, ARSEF549 and BRIP53293, and *M. brunneum* ARSEF3297 (Figure  4). Cytochrome P450s are a group of proteins that play a role in several metabolic processes, such as primary metabolism, membrane ergosterol biosynthesis, SMs synthesis, and detoxification of harmful compounds. We identified 117 genes in *M. humberi* ESALQ1638 associated with cytochrome P450s, compared to 120 genes in *M. robertsii* ARSEF23, 109-116 genes in *M. anisopliae* isolates, and 116 genes in *M. brunneum* ARSEF3297. Glycoside hydrolases (GHs) are enzymes that catalyze the hydrolysis of glycosidic bonds in sugars. They are involved in the degradation of biomass, pathogenesis mechanisms, and several cellular functions associated with basal metabolism. We found 91 predicted protein-coding genes in *M. humberi* ESALQ1638 related to the GH interpro category compared to 101 in *M. robertsii* ARSEF23, 97–101 in *M. anisopliae* isolates, and 89 in *M. brunneum* ARSEF3297. Interestingly, within the *Metarhizium* species, the intermediate host range strains *M. guizhouense* ARSEF977, and *M. majus* ARSEF297 showed the highest number of predicted protein-coding genes associated with cytochrome P450s, GH, P-loop containing nucleoside triphosphate hydrolases, and fungal transcription factors. Nonetheless, the phytopathogen *F. verticillioides* and the mycopathogen *T. harzianum* showed a higher number of genes related to GH with 152 and 141, respectively. Major facilitator transporters (MFTs) are membrane proteins associated with the transport of various substrates, such as monosaccharides, amino acids, vitamins, enzyme cofactors, anions, and cations. We found a similar number of predicted protein-coding genes associated with MFT in *M. humberi* ESALQ1638 (314), *M. robertsii* ARSEF23 (313), *M. anisopliae* isolates (305–313), and *M. brunneum* ARSEF3297 (301).

The fungi *A. niger*, *F. verticillioides*, and *T*. *harzianum* showed more predicted protein-coding genes for almost all selected InterPro categories. In comparison, the narrow-host range *M. album* ARSEF1941 and *M. rileyi* RCEF4871 had a lower number of predicted-protein genes assigned in all InterPro categories (Figure  4).

In contrast with what was found in the first investigation (Figure  4), the broad and intermediate host range *Metarhizium* species showed a higher number of predicted protein-coding genes in these InterPro categories compared to outgroups. Specifically, the broad host rage species, *M. humberi* ESALQ1638, *M. robertsii* ARSEF23, *M. anisopliae* ESALQE6, ARSEF549, and BRIP53293, and *M. brunneum* ARSEF3297 and the intermediate host range strains *M. majus* ARSEF297, and *M. guizhouense* ARSEF977 had similar numbers of predicted coding-protein genes. In contrast, the narrow host range strains *M. acridum* CQMa102, *M. album* ARSEF1941, and *M. rileyi* RCEF4871 had fewer genes in all InterPro categories (Figure  5). We found a high number (*n* = 72) of predicted protein-coding genes associated with an acyl carrier protein (ACP-like superfamily) in the *M. humberi* ESALQ1638 genome. ACP-like protein plays a role in the synthesis of fatty acids in bacteria and plants and polyketide biosynthesis. In addition, we highlight that *M. humberi* ESALQ1638 had a higher number of genes associated with the enzymes PKS and dehydratase domain (*n* = 36) compared to *M. robertsii* (*n* = 27), *M. anisopliae* (*n* = 28–29), and other *Metarhizium* spp. (ranged from 10 to 29) (Figure  5).

### Genomic signatures of *Metarhizium humberi* ESALQ1638

**Figure 6:**
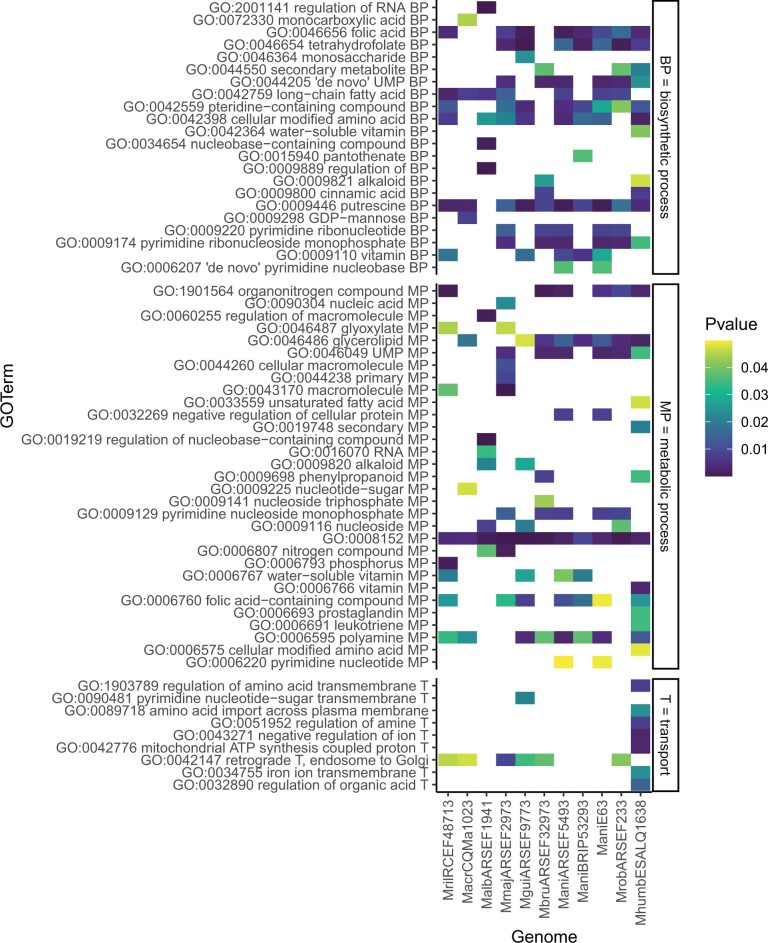
Selected enriched Gene Ontology terms (Biological Process) in rapidly evolving gene families of 11 *Metarhizium* genomes belonging to eight species. Shown are all significantly overrepresented Biological Process GO terms that contained the terms “biosynthetic process” (BP, top), “metabolic process” (MP, middle), and “transport”/“import”/“export” (T, bottom). The *P*-value for each GO term and genome is indicated by color; the cutoff for significance was 0.05. A plot containing all overrepresented GO terms from all three ontology categories can be found on the Gitlab repository associated with this publication: https://gitlab.nibio.no/simeon/iwanicki_et_al_21/-/tree/master/Cafe_Aug21.

Our previous results from this article showed that the genome of *M. humberi* ESALQ1638 has several similarities with *M. anisopliae* isolates and *M. robertsii* ARSEF23. Here, we aimed to elucidate the genomic signatures of *M. humberi* ESALQ1638 by contrasting overrepresented GO-terms in biological process category in rapidly evolving gene families in *Metarhizium* species and highlighting the unique ones in *M. humberi* ESALQ1638. We identified 188 overrepresented GO-terms in rapidly evolving genes families at a *p-value* ≤0.05 across the *Metarhizium* species (Additional file 11). Of this total, 83 represents biological process, 35 cellular components, and 70 molecular functions. Within the biological process category, we found that seven out of 16 unique *M. humberi* ESALQ1638 overrepresented GO-terms are associated with transport of amino acid (GO: 0051952, GO: 1903789, GO: 0089718), ions (GO: 0034755, GO: 0042776, GO: 0043271), or organics acid (GO: 0032890) (Figure  6). Besides these GO-terms, we highlight the unique overrepresented GO-terms in *M. humberi* ESALQ1638 associated to secondary metabolic process (GO: 0019748), unsaturated fatty acid metabolic process (GO: 0033559), water-soluble vitamin biosynthetic process (GO: 0042364) and vitamin metabolic process (GO: 0006766), meiotic chromosome segregation (GO: 0045132) and RNA polymerase III preinitiation complex assembly (GO: 0070898) (Figure  6 and Additional file 11). Conversely, we found three unique overrepresent GO-terms in *M. robertsii* associated with DNA activities (GO: 0003678: DNA helicase activity, GO: 0005657: replication fork, GO: 0032508: DNA duplex unwinding) and no unique overrepresent GO-terms in *M. anisopliae*. The only overrepresent GO-term of biological process in all three *M. anisopliae* isolates (ESALQE6, ARSEF549, and BRIP53293) that was not overrepresented in *M. humberi* proteome is the vitamin biosynthetic process (GO: 0009110).

On the other hand, the SM biosynthetic process GO: 0044550 GO-term was overrepresented in *M. humberi*, *M. robertsii*, and *M. brunneum* in rapidly evolving gene families. In contrast, the pathogenesis GO-term GO: 000940) was overrepresented in *M. acridum*, *M. robertsii*, *M. humberi*, *M. majus*, *M. guizhouense*, and *M. brunneum.* Interestingly, the only GO-term from the biological process category not overrepresented in species with narrow (*M. rileyi*, *M. album*, and *M. acridum*) and intermediate host range (*M. guizhouense* and *M. majus*) but overrepresented in species with broad host range (*M. anisopliae*, *M. robertsii* and *M. humberi*) is associated to organonitrogen compound metabolic process (GO: 1901564) (Additional file 10). Considering the overrepresented GO-term in molecular function category in *M. humberi*, we highlight the serine-type peptidase activity (GO: 0008236), protein tyrosine/serine/threonine phosphatase activity (GO: 0008138), kinase activity (GO: 0016301), and metallopeptidase activity (GO: 0008237) (Additional file 11). A plot containing all overrepresented GO-terms from all three ontology categories can be found on the Gitlab repository associated with this publication: https://gitlab.nibio.no/simeon/iwanicki_et_al_21/-/tree/master/Cafe_Aug21.

### Secretome analysis

We predicted the secretome of *M. humberi* ESALQ1638, *M. anisopliae* ARSEF549, *M. robertsii* ARSEF23, and *M. rileyi* RCEF4871 by combining a set of bioinformatics tools. Results showed that 706, 752, 774, and 457 proteins are predicted to be secreted, representing 6.6%, 6.6%, 6.6%, and 5.2% of the complete *M. humberi* ESALQ1638, *M. anisopliae* ARSEF549, *M. robertsii* ARSEF23, and *M. rileyi* RCEF4871 proteome, respectively. Interestingly, we found that *M. humberi* proteome is composed of a lower percentage of coding sequences associated with glycoside hydrolase and carbohydrate-binding modules than *M. anisopliae*, *M. robertsii*, and *M. rileyi* proteome (Table  4 and Additional file 12). On the other hand, *M. humberi* proteome has a slightly higher number of carbohydrate esterases than in *M. anisopliae*, *M. robertsii*, and *M. rileyi* proteomes (Table  4).

**Table 4:** Total number of secretome proteins and profiles of CAZymes in the secretomes of *M. rileyi* RCEF4871, *M. robertsii* ARSEF23, *M. humberi* ESALQ1638, and *M. anisopliae* ARSEF549

	*M. rileyi*	*M. humberi*	*M. anisopliae*	*M. robertsii*
No of secretome proteins	457 (5.215)	706 (6.640)	752 (6.582)	774 (6.622)
Glycoside hydrolase	74 (0.844)	73 (0.687)	92 (0.805)	98 (0.838)
Auxiliary activities	23 (0.262)	35 (0.329)	37 (0.324)	35 (0.299)
Carbohydrate-binding modules	7 (0.080)	3 (0.028)	10 (0.088)	8 (0.068)
Carbohydrate esterase	5 (0.057)	8 (0.075)	6 (0.053)	7 (0.060)
Glycosyltransferase	1 (0.011)	1 (0.009)	1 (0.009)	1 (0.009)
Polysaccharide lyase	0 (0.000)	3 (0.028)	3 (0.026)	2 (0.017)

The values in brackets indicate percentage of coding sequences in genome.

Fungi secrete a high diversity of carbohydrate-active enzymes (CAZymes), which reflect an ability to metabolize different substrates and explore new niches. In comparison with narrow the host range species *M. rileyi*, the broad host range species *M. humberi*, *M. robertsii*, and *M. anisopliae* have a more significant number of enzymes in their proteomes related to the degradation of chitin (GH18) and chitosan (GH75, GH2), which are the main components of the integument of arthropods, and lysozymes (GH24 and GH25) that act in the lysis of carbohydrates, such as the peptidoglycan component of the bacterial cell wall. In addition, *M. humberi* ESALQ1638 has a greater number of enzymes related to the degradation of chitin (CH18) and the degradation of xylan (GH3 and CE3), which is a hemicellulose component present in plants, compared to *M. anisopliae* and *M. robertsii* (Table  5).

**Table 5:** Enzyme annotation overview for *M. rileyi* RCEF4871, *M. robertsii* ARSEF23, *M. humberi* ESALQ1638, and *M. anisopliae* ARSEF549 in relation to action on the substrate cellulose, xylan, starch, chitin, chitosan, galactose, trehalose, peptidoglycan, and slycosyl compounds

Substrate	Enzyme code (EC)	CAZymes	*M. rileyi*	*M. humberi*	*M. robertsii*	*M. anisopliae*
Cellulose	3.2.1.4	GH12	*	*	–	–
Cellulose	3.2.1.23	GH35	–	**	*	*
Chitin	3.2.1.14	GH18	–	******	***	**
Chitosan	3.2.1.132	GH75	–	**	***	**
Chitosan or chitosan oligosaccharides	3.2.1.165	GH2	*	*	*	*
Galactose	3.2.1.22	GH36	*	–	*	*
Glycosyl compounds	3.2.1.113; 3.2.1.24	GH47	*	**	**	*
Glycosyl compounds	3.2.1.51	GH29	*	*	*	*
Glycosyl compounds	3.2.1.3	CBM20	*	–	*	*
Glycosyl compounds	3.2.1.55	CBM42	*	–	*	*
Glycosyl compounds	3.2.1.52	GH20	*	–	*	*
Glycosyl compounds	3.2.1.55	GH54	*	–	*	*
Glycosyl compounds	3.2.1.101; 3.2.1.24	GH76	*****	**	*****	**
Pectate and other galacturonans	3.2.1.15	GH28	**	*	*	*
Peptidoglycan/chitodextrins	3.2.1.17	GH24	–	**	**	**
Peptidoglycan/chitodextrins	3.2.1.17	GH25	–	**	**	**
Strach	3.2.1.1	GH13_1	*	–	*	–
Strach	3.2.1.3	GH15	*	–	*	*
Trehalose	3.2.1.28	GH37	–	*	–	*
Xylan	3.4.24	CE3	–	*	–	–
Xylan	3.2.1.21	GH3	*	**	**	*
	1.1.3.4; 1.1.3.5	AA3_2	–	*	–	–
	1.11.1.7	AA2	**	**	***	**

Asterisks indicate the number of genes in genome associated with the specific enzyme. AA, auxiliary activities; CB, carbohydrate-binding modules; CE, carbohydrate esterase; GH, glycoside hydrolase.

In *M. humberi* ESALQ1638, gene ontology groups could be assigned to 60.0% (423/706) of the secretome and classified into eight groups in the biological process domain and 14 groups in the molecular function, and four groups in the cellular component domain. The largest set of secreted proteins was assigned to proteolysis (GO: 0006508), oxidation-reduction process (GO: 0055114), and oxidoreductase activity (GO: 0016491; GO: 0016614), representing 29% (204/706) of the secretome (Additional file 12). Protein family domains could be assigned to 53.0% (374/706) of the complete *M. humberi* ESALQ1638 secretome. According to the gene ontology analysis, the most extensive set of secreted proteins could be assigned to the subtilases (PF00082), aspartyl proteases (PF00026), trypsins (PF00089; PF13365), and multicopper oxidases (PF00394; PF07731; PF07732), representing 12.5% (89/709) of the secretome (Additional file 12).

*Metarhizium* *humberi* ESALQ1638 possesses an arsenal of enzymes representing 23.6% (167/706) of the secretome. We found 218 enzymes (EC numbers assigned) related to 167 secreted proteins, represented mainly by hydrolases that act on peptide bonds 46.7% (102/218), including EC 3.4.21, EC 3.4.23 and EC 3.4.16, carboxylesterase (EC 3.1.1.1) 7.0% (15/218), triacylglycerol lipases (EC 3.1.1.3) 4.5% (10/218) and chitinases (EC 3.2.1.14) 3.2% (7/218) (Additional file 12).

### Serine peptidases

Fungal SPs are involved in symbiotic or pathogenic interactions with plant hosts, insect hosts, nematode hosts, and other fungi (Reddy *et al*. 1996). We showed that *M. humberi* ESALQ1638 has one overrepresented gene ontology term in rapidly evolving gene families associated with SPs (GO: 0008236). In addition, the secretome results showed a high number of enzymes related to peptidases. Here, we investigated the SP families present in *M. robertsii* ARSEF23, *M. anisopliae* BRIP53293, and *M. humberi* ESALQ1638.

SPs were grouped into 15 families, among which S09 had the highest number of proteins. S09 peptidases are associated with protein maturation, alpha-factor maturation, extracellular degradation, and vacuole protease (Table  6). *Metarhizium* *humberi* ESALQ1638 showed a slightly higher number of S09 peptidases (*n* = 178) compared to *M. robertsii* ARSEF23 (*n* = 174) and *M. anisopliae* BRIP53293 (*n* = 174). The second and third most common families of peptidases are S33, which is not well studied in fungi, and S08, associated with extracellular degradation, intracellular prohormone activation, and intracellular protein degradation (Table  6). *Metarhizium* *humberi* ESALQ1638 has similar numbers of S033 (*n* = 60) and S08 (*n* = 54) compared to *M. robertsii* ARSEF23 (S033 = 60; S08 = 56) and *M. anisopliae* BRIP53293 (S033 = 62; S08 = 50). The three SP families in which *M. humberi* ESALQ1638 has more peptidase-related genes than *M. robertsii* ARSEF23 and *M. anisopliae* BRIP53293 are S09, S15, with unknown function in fungi; and S53, associated with TppI—lysosomal enzyme, degradation of extracellular proteins (Table  6).

**Table 6:** Main serine peptidase families present in fungi and number of proteins in *M. humberi* ESALQ1638, *M. robertsii* ARSEF23, and *M. anisopliae* BRIP53293

Serine peptidase family (MEROPS ID)	*M. humberi*	*M. robertsii*	*M. anisopliae*	Functions
S01	36	36	37	Chaperone, extracellular degradation
S08	54	56	50	Extracellular degradation, intracellular prohormone activation, intracellular protein degradation
S09	178	174	174	Protein maturation, alpha factor maturation, extracellular degradation, vacuole protease
S10	11	11	11	Vacuole protease, extracellular degradation
S12	20	20	22	Chitin degradation
S14	1	1	1	Mitochondrial protein involved in protein maturation and stress reaction
S15	4	2	2	Unknown in fungi
S16	7	7	7	Misfolded protein degradation in mitochondria
S26	2	2	3	Maturation of mitochondrial proteins
S28	5	5	5	Unknown in fungi
S33	60	60	62	Understudied in fungi, yeast proteins similar to proline proteases
S49	14	15	14	Unknown in fungi
S53	10	9	9	TppI—lysosomal enzyme, degradation of extracellular proteins
S54	28	28	27	Mitochondrial endopeptidase
S59	1	1	1	Essential for nuclear pore formation

Functional annotation was extracted from Muszewska *et al.* (2017) and summarized based on SGD (Cherry *et al.* 2012) functional annotation and literature searches.

### Secondary metabolites

We identified *M. humberi* ESALQ1638 proteins involved in SMs' biosynthesis, such as PKSs, NRPSs, and hybrid NPKS-PKS. We found 47 proteins associated with PKSs in the *M. humberi* ESALQ1638 genome, 23 of which had high sequence similarity to PKSs identified and described by Donzelli and Krasnoff (2016) in the *M. anisopliae* ARSEF459 genome (Additional file 13). Some of those PKSs have known products, such as aurovertins, which inhibit mitochondrial, bacterial, and chloroplast ATPases (F1) and act by uncoupling oxidative phosphorylation (Mao *et al.* 2015). This PKS is not present in the genomes of *M. acridum* CQMa102, *M. album* ARSEF1941, *M. majus* ARSEF297, and *M. rileyi* RCEF4871. The pathway for viridicatum toxin was shared with all *Metarhizium*, except *M. rileyi* RCEF4871; viridicatum toxin was identified as a tetracycline‐like antibiotic. Three PKSs (MHUMG1_08916, MHUMG1_10616, MHUMG1_06663) without sequence similarity with genes identified by Donzelli and Krasnoff (2016) are unique to *M. humberi* ESALQ1638 (Additional file 13). A total of 36 and 35 PKSs are shared with *M. robertsii* ARSEF23 and *M. anisopliae* ARSEF549, respectively, while only ten are shared with *M. rileyi* RCEF4871. We found 13 proteins identified as NRPSs in *M. humberi* ESALQ1638 (Additional file 13). Destruxin is a typical NRPS *Metarhizium* metabolite associated with virulence toward insect hosts (Wang *et al.* 2012), and pathways for it were found in all *Metarhizium* except *M. album* ARSEF1941, *M. acridum* CQMa102, and *M. rileyi* RCEF4871. Ferricrocin (M‐NRPS17) and metachelin (M‐NRPS18) are both siderophores that are small molecules produced by plants and microorganisms with high binding affinity and iron selectivity (Sbaraini *et al.* 2017). Both M‐NRPS17 and ‐18 are genomic regions conserved in all *Metarhizium* strains, except for *M. album* ARSEF1941, which lacks genes involved in the biosynthesis of Metachelin. We found seven proteins in *M. humberi* ESALQ1638 associated with NRPS-PKS. The M-HPN1 (MHUMG1_1965) was identified by Donzeli and Krasnoff (2016), a xenolozoyenone-related compound (Sbaraini *et al.* 2017); the M-HPN4 (MHUMG1_7413) and the M-HPN6 (MHUMG1_8807). Four (MHUMG1_3, MHUMG1_3562, MHUMG1_9424 and MHUMG1_9818) proteins has no homologs identified by Donzeli and Krasnoff (2016) in other *Metarhizium* spp.

### Proteins involved in PHI

We identified 1969 proteins (18.5% of the *M. humberi* ESALQ1638 genome) similar to experimentally verified proteins that play a role in PHI in other fungi, mostly plant pathogens, in plant-pathogenic bacteria, insect and mite pathogen, and endophytes (Additional file 16). We found 40 orthologous proteins associated with increased virulence toward arthropods in *M. humberi* ESALQ1638, 44 in *M. anisopliae* BRIP53293, and 39 in *M. robertsii* ARSEF23 genome (Additional file 14). The functions of these proteins are very similar among the three *Metarhizium* species. We highlight that while *M. humberi* has three endochitinases, *M. anisopliae* BRIP53293 and *M. robertsii* ARSEF23 have six, all orthologous to *M. anisopliae* BRIP53293. *Metarhizium* *humberi* ESALQ1638 has one dehydrogenase (*PHI: 6957*), one fatty acid oxygenase (*PHI: 494*) and one part of an NADPH oxidase complex (*PHI: 3934*), orthologous to *X. oryzae* (a plant pathogenic bacteria), *A. nidulans* (a saprophytic fungus used for studying eukaryotic cell biology), and *C. purpurea* (a plant pathogenic fungus), respectively, and are not found in the *M. robertsii* ARSEF23 genome. Conversely, *M. humberi* ESALQ1638 has one protein assigned to indole-3-acetic acid (IAA) biosynthesis (*PHI: 6650*), which was also found in *M. robertsii* ARSEF23 but not in *M. anisopliae* BRIP53293. The only protein in the *M. humberi* ESALQ1638 genome associated with virulence and not found in *M. robertsii* ARSEF23 and *M. anisopliae* BRIP53293 was a PKS (*PHI: 5038*), which is orthologous to one PKS from the fungus *B. bassiana*. Some genes in the PHI database are classified as effector genes, formerly known as avirulence genes. Effectors either activate or suppress plant defense responses (Urban *et al.* 2015). We identified 21 effectors in *M. humberi* ESALQ1638, 18 in *M. anisopliae* BRIP53293, and 20 in *M. robertsii* ARSEF23. Eight effectors in *M. humberi* ESALQ1638 are glucanase inhibitor proteins (*PHI: 653*), while seven and nine were found in *M. robertsii* ARSEF23 and *M. anisopliae* BRIP53293, respectively. Five effectors in *M. humberi* ESALQ1638 and *M. robertsii* ARSEF23 are associated with PKSs (*PHI: 325*), while three were found in *M. anisopliae*. We want to highlight that *M. humberi* ESALQ1638 has one effector (*PHI: 981*), orthologous with a protein in *Pseudomonas syringae* (a plant pathogenic bacteria), that is not present in *M. robertsii* ARSEF23 or *M. anisopliae* BRIP53293 (Additional file 14).

## Discussion

In this study, we describe the genome of *M. humberi* ESALQ1638 recovered from Brazilian soil of native vegetation, previously described as a plant root symbiont and insect and mite-pathogenic fungus (Castro *et al.* 2018; Luz *et al.* 2019; Riguetti Zanardo Botelho *et al.* 2019; Siqueira *et al.* 2020). The *M. humberi* ESALQ1638 genome has an equal number of orthologous genes shared with the broad insect-host range and symbiont species *M. anisopliae* and *M. robertsii*, but fewer orthologs compared to the narrow insect-host range strains *M. album* ARSEF1941, *M. rileyi* RCEF4871, and *M. acridum* CQMa102. We identified overrepresented biological process GO-terms in rapidly evolving gene families in *M. humberi* ESALQ1638 and an arsenal of enzymes in its secretome that may be associated with the ability to expand to new host niches or infect different orders of insects. These include biological process associated to SMs, transport of amino and organic acid and carbohydrate active enzymes (CAzymes) that act on different substrates such as chitin, chitosan and trehalose, from arthropods, and cellulose, peptidoglycan, and xylan from plants. Indeed, *M. humberi* can explore different niches, such as free-living in soil (Rezende *et al.* 2015; Iwanicki *et al.* 2019; Riguetti Zanardo Botelho *et al.* 2019), infecting diverse orders of insects (Lopes *et al.* 2014; Rezende *et al.* 2015; Brunner-Mendoza *et al.* 2017) and establishing associations with plants (Canassa *et al.* 2019c; Lira *et al.* 2020; Siqueira *et al.* 2020) and should be considered a broad host range species. On the other hand, narrow host range species associated only with specific insect orders are found at lower frequencies in soil and associated with plants. The ability to expand to new host niches is a characteristic also shared with *M. anisopliae* and *M. robertsii* (Lopes *et al.* 2014; Rezende *et al.* 2015; Iwanicki *et al.* 2019; Riguetti Zanardo Botelho *et al.* 2019), the fact that likely explains the high similarity found between genome sequences and functionality of genes in *M. humberi*, *M. anisopliae*, and *M. robertsii*.

*Metarhizium* live in different host niches, and this versatility is associated with their ability to secrete many enzymes that degrade substrates available in the growing environment and absorb degraded compounds (Staats *et al.* 2014). We identified in *M. humberi* ESALQ1638 a most extensive set of secreted proteins assigned to proteolysis. Specifically, SPs were overrepresented in rapidly evolving genes families in *M. humberi* ESALQ1638 and in *M. anisopliae* ARSEF549, ESALQE6 and *M. brunneum* ARSEF32973. These enzymes are associated with several functions in fungi, plants, and protozoa, such as immune response, signal transduction, protein maturation, virulence, and nutrient breakdown and acquisition (Rawlings and Barrett 1993; Muszewska *et al.* 2017). One well-studied SP involved in pathogenicity and root colonization by *M. robertsii* and *M. anisopliae* and with orthologs in *M. humberi* ESALQ1638 is the subtilisin-like SP Pr1A. This enzyme is expressed during early infection in insect cuticles (Javar *et al.* 2015) and is highly expressed during root colonization (Pava-Ripoll *et al.* 2011; Moonjely and Bidochka 2019). We investigated families of SP s in the *M. humberi* ESALQ1638 genome that might be associated with penetration and colonization of plant and/or insect hosts. Although similar numbers of genes were found in serine-peptidase families in *M. robertsii* ARSEF23 and *M. anisopliae* BRIP53293, *M. humberi* ESALQ1638 has a slightly higher number of SPs from families S09 (prolyl oligopeptidase), S15 (X-Pro-dipeptidyl-peptidase), and S53 (sedolisin). In other functions, prolyl oligopeptidase and sedolisin are associated with the degradation of extracellular proteins (Reichard *et al.* 2006; Muszewska *et al.* 2017), including insect cuticles and plant epidermal proteins. Sedolisins secreted by *A. fumigatus* were shown to degrade proteins at low pH values and generate assimilable nitrogen sources to decompose organic matter and composts (Reichard *et al.* 2006). The high number of sedolisins in *M. humberi* ESALQ1638 might be associated with these proteins' involvement in saprophytic and symbiotic lifestyles, and their functions are worth investigating in the future. Nonetheless, we determined that compared with noninsect-pathogenic fungi, *A. niger*, *F. verticillioides*, and *T. harzianum*, generalist and intermediate host range *Metarhizium* spp. have more genes in all interpro categories associated with subtilisin peptidases (IRP037045, IRP009003, IRP015500, and IRP036852). Bagga *et al.* (2004) suggested that a high number of subtilisin peptidases in *Metarhizium* sp. is associated with pathogenicity to insects and increased adaptability and host range and might play a role in survival in various ecological habitats outside the host.

In addition to the subtilisin-like SP Pr1A, other experimentally characterized genes involved in pathogenicity and root colonization and present in *M. humberi* ESALQ1638, *M. robertsii* ARSEF23, *M. anisopliae* BRIP53293, and *M. brunneum* ARSEF3297 genomes are the insect cuticle binding adhesin Mad1 and the plant epidermis binding adhesin Mad2 (Wang and St. Leger 2007). Adhesins are cell wall proteins that play a role in the first step of host interaction. This protein anchors fungal conidia to arthropod and plant surfaces, enabling the fungus to persist and colonize these different hosts effectively. Disruption of Mad 1 or Mad 2 produced an approximately 90% reduction in adherence of *M. anisopliae* conidia to locust cuticle and fly wings and onion and celery epidermis (Wang and St. Leger 2007), indicating the extreme importance of these proteins in the first step of insect/plant colonization.

The fungus secretes several enzymes involved in nitrogen and carbon acquisition during host colonization, critical nutrients for biomass production. Behie *et al.* (2017) demonstrated the ability of *M. robertsii* to establish a symbiotic relationship with haricot bean roots, as it provides accessible carbons to the fungus in exchange for insect-derived nitrogen and suggested the involvement of nutrient transporters. In this study, we found that seven out of 16 unique *M. humberi* ESALQ1638 biological process overrepresented GO-terms in rapidly evolving genes families are associated with transport of amino ions, organics, and amino acids. Amino acid permeases and ammonium permease are within the primary nitrogen transporters in fungi (Holsbeeks *et al.* 2004; Walker and White 2005). Moonjely and Bidochka (2019) assessed the role of six genes of *M. robertsii* in endophytic, rhizoplane, and rhizospheric colonization of barley roots. The authors found that the deletion of two ammonium permeases, MepC and Mep2 of the *M. robertsii* genome, enhanced rhizoplane colonization and promoted higher nitrogen incorporation of insect-derived nitrogen in barley leaves. In the *M. humberi* ESALQ1638 genome, we found orthologous genes for each permease MepC and Mep2, which were also present in *M. brunneum* ARSEF3297 and all three *M. anisopliae* strains: ESALQE6, BRIP53293, and ARSEF549. This suggests that *M. humberi*, *M. brunneum*, and *M. anisopliae* have the same mechanism as *M. robertsii* to mobilize nitrogen during plant interactions. Besides that, our results showed that the only overrepresented GO-term in common in rapidly evolving gene families of *M. anisopliae*, *M. robertsii*, *M. humberi* proteome is the organonitrogen compound metabolic process. This result suggests the expansion of host range in *Metarhizium* is driven by a positive selection pressure associated to the metabolism of organic nitrogen compounds delivered from different substrates.

While plants benefit from assimilable forms of nitrogen provided by symbiotic fungi, the fungus absorbs carbon presented in photosynthate in the form of several types of sugar (Rovira 1969). Raffinose is a trisaccharide composed of galactose, glucose, and fructose, which occur mainly in seeds, roots, root exudates, and underground stems with the likely carbohydrate reserve function (Rovira 1969). We identified a raffinose transporter (Mrt) in *M. humberi* ESALQ1638 orthologous to *M. anisopliae* and *M. robertsii*. Mrt is essential for fungi to grow on raffinose family oligosaccharides, and orthologous to this gene in *M. robertsii* was shown to be exclusively involved in interactions with plants (Fang and St. Leger 2010). It is worth mentioning that the opportunistic fungus *A. niger* and the plant pathogenic and endophytic *F. verticillioides* have two and three times the number of genes associated with sugar transport, respectively (interpro category: IPR003663 and IPR005828), compared to *Metarhizium* spp., which suggests the expanded ability of those species to assimilate sugar from plants.

Besides expanding nutrient uptake and translocation by plants, endophytic/entomopathogenic fungi can enhance plant growth by modulating phytohormones involved in growth and development (Hu and Bidochka 2019). Indole-3-acetic acid (IAA) is an auxin class phytohormone associated with cell division and elongation induction. The production of auxins by *M. anisopliae*, *M. robertsii*, and *M. brunneum* has been demonstrated in previous studies (Liao *et al.* 2014). *Metarhizium* *humberi* ESALQ1638 has one protein assigned to indole-3-acetic acid (IAA) biosynthesis (PHI: 6650), which is also found in *M. robertsii* ARSEF23, not in *M. anisopliae* BRIP53293, suggesting that the production of this phytohormone by *Metarhizium* might be isolate-specific. Another phytohormone involved in plant growth and development and resistance to abiotic and biotic stress is salicylic acid (SA) (Maruri-López *et al.* 2019). During pathogenic interactions between plants and plant pathogens, SA accumulates in the local infected tissue and then spreads throughout the plant to induce systemic acquired resistance at noninfected distal parts of the plant (Maruri-López *et al.* 2019). Interestingly, we identified one effector (*PHI: 981*) in the *M. humberi* ESALQ1638 genome, orthologous to the protein HopI1 in *P. syringae* (plant pathogenic bacteria), not present in the *M. robertsii* ARSEF23 or *M. anisopliae* BRIP53293 genome. Effectors are molecules known to activate or suppress plant defense responses (Urban *et al.* 2015). The HopI1 effector was shown to suppress SA accumulation inside the plant (Jelenska *et al.* 2007). Similar to the function in *P. syringae*, the expression of genes orthologous to Hop1 in *M. humberi* ESALQ1638 might be a strategy to avoid activating the plant defense system during symbiotic interactions with plants. Another plant defense response against fungal invasion is the production of enzymes that degrade polysaccharides from the fungal cell wall, such as endo-β-1,3-glucanases (Rose *et al.* 2002). In response to this defense, pathogens secrete glucanase inhibitor proteins (GIPs), which inhibit the endoglucanase activity of their plant host (Kamoun 2003). We found eight effectors in *M. humberi* ESALQ1638 identified as GIPs (*PHI: 653*), while seven and nine were found in *M. robertsii* ARSEF23 and *M. anisopliae* BRIP53293, respectively. GIPs are well studied in plant pathogenic fungi from the genus *Phytophthora* (Rose *et al.* 2002).

SM produced by fungal symbionts and endophytes play an essential role in inducing plant defense and virulence, enhancing the efflux of nutrients by plants, protecting the host from pests and diseases, and mediating communications with the plant host (Mukherjee *et al.* 2018; Lugtenberg *et al.* 2016; Björkman *et al.* 2011). Examples are peramine, an insect feeding deterrent produced by the grass endophyte *Epichlo efestucae*, and siderophores ferricrocin and metachelin (Koulman *et al.* 2007). The first class of SMs we investigated here is polyketides. We found a higher number of PKS encoding genes in the *M. humberi* ESALQ1638 genome than in other *Metarhizium*, some of which are associated with virulence toward arthropods. The only protein in the *M. humberi* ESALQ1638 genome orthologous to the PHI database associated with virulence and not found in *M. robertsii* and *M. anisopliae* was a PKS (*PHI: 5038*), which is orthologous to oosporein synthase 1 from the entomopathogenic fungus *B. bassiana*. Oosporein is a well-studied toxin produced by several endophytic fungi and plant- and insect pathogenic fungi with an array of biological activities (Feng *et al.* 2015). In *B. bassiana*, this toxin has an immunomodulation property (Mc Namara *et al*. 2019), but it has not been reported in the *Metarhizium* genus before (Hu *et al.* 2014; Feng *et al.* 2015). Therefore, our investigation is the first evidence that *Metarhizium* might also produce oosporein; experimental studies are required to confirm this hypothesis. In addition, we found several PKSs with no orthologs identified by Donzelli and Krasnoff (2016) and three PKSs with no orthologs in other *Metarhizium*. The role of those PKSs in *M. humberi* lifestyle remains unknown.

The second group of SMs investigated here was NRPSs. In contrast with the high number of PKSs in *M. humberi* ESALQ1638, we found a similar number of genes that encode NRPSs in *M. humberi* ESALQ1638 compared with broad and intermediate host-range *Metarhizium* spp. Three of them are known to play a pivotal role in the *Metarhizium* lifestyle: Destruxins (M-NRPS19), Ferricrocin (M‐NRPS17), and Metachelin (M‐NRPS18). Destruxins suppress host defenses in insects during fungal infection (Lu and St. Leger 2016), while ferricrocin and metachelin are intra- and extracellular siderophores, respectively. They enhance iron acquisition by fungi and involve infecting insects and scavenging extracellular iron in a saprophytic lifestyle (Donzelli *et al.* 2016). Mutating a ferricrocin gene showed its virulence role due to delayed germination and alterations in endogenous fungal iron content (Donzelli *et al.* 2015).

The *M. humberi* ESALQ1638 genome had fewer retroelements and DNA transposons than the genomes of *M. robertsii* and *M. anisopliae*. Transposable elements (TEs) are DNA sequences that move from one location on the genome to another by a “copy and paste” or “cut and paste” mechanism (Pray 2008). To address transposons, fungi have an exclusive genomic defense mechanism called RIP, which inactivates transposon activity and its replication in the genome. The high RIP amount in *M. humberi* might explain the lower number of TEs in its genome than *M. robertsii* and *M. anisopliae*. Significantly higher RIP regions are present in narrow host-range species such as *M. acridum* and *M. album* but are almost absent in broad host-range species (Gao *et al.* 2011; Hu *et al.* 2014).

Nonetheless, RIP only functions during meiosis, and its presence in genomes of narrow host-range species is associated with retention of sexual reproduction (Gao *et al.* 2011; Hu *et al.* 2014; Wang *et al.* 2016). A recent study on *Metarhizium* diversity in soil from Brazilian biomes showed that *M. humberi* isolates (described in the publication as *Metarhizium* sp. indet. 1) have higher haplotype and nucleotide diversity of the MzIGS3 region compared to *M. robertsii* and *M. anisopliae* isolates (Riguetti Zanardo Botelho *et al.* 2019). Although the presence of TEs in the genome is frequently associated with genome diversity, we believe that in *M. humberi*, the retention of some sexual reproduction activity might be related to genome diversity found in Riguetti Zanardo Botelho *et al.* (2019).

Our genome analysis of the newly described species *M. humberi* showed that the analyzed isolate ESALQ1638 shared many characteristic traits with other closely related species within the *Metarhizium* PARB clade. These included a similar size genome, an equal number of secreted enzymes, a more significant number of enzymes related to degradation of chitin and plant cell wall, overrepresented GO-terms in rapidly evolving genes families associated to SM biosynthetic process and organonitrogen compound metabolic process, and NPRSs. However, the genome of *M. humberi* ESALQ1638 also revealed some unique traits that stood out, *e.g.*, more genes functionally annotated as PKSs, overrepresented GO-terms associated with ions transport, organics and amino acid, a higher percentage of repetitive elements, and higher levels of RIP-induced point mutations. Thus, the genomic data supported the broad host range of this species within the generalist PARB clade and suggested that *M. humberi* ESALQ1638 might be particularly good at producing SMs and might be more efficient in transporting amino acids and organics compounds. However, we recognize that future genomic studies with more *M. humberi* isolates will be necessary for a better understanding of the genomic signatures of this species since there are a great diversity of biological responses among isolates of *Metarhizium* species.

## Data availability

This Whole Genome Shotgun project has been deposited at DDBJ/ENA/GenBank under the accession JACEFI000000000. The version described in this paper is version JACEFI010000000. Custom scripts for phylogenetic tree generation, Mummer-like plotting, and CAFE5/Gostats analysis have been publicly available through the GitHub repository: https://gitlab.nibio.no/simeon/iwanicki_et_al_21. The additional files are deposited at GSA figshare portal: https://doi.org/10.25387/g3.14489004.
